# MiR-214 sensitizes human colon cancer cells to 5-FU by targeting Hsp27

**DOI:** 10.1186/s11658-019-0143-3

**Published:** 2019-03-14

**Authors:** Yong Yang, Yan Bao, Guo-Kai Yang, Jia Wan, Ling-Juan Du, Zhen-Huan Ma

**Affiliations:** 1 The Third Department of General Surgery, The Second People’s Hospital of Yunnan Province, Kunming, China; 2 Department of Vascular Surgery, The Fourth Affiliated Hospital of Kunming MedicalUniversity, 176 Youth Road, Kunming, Yunnan Province 650021 People’s Republic of China

**Keywords:** miR-214, Hsp27, 3′-UTR, 5-FU, Colon cancer

## Abstract

Overcoming chemorestistance to 5-fluorouracil (5-FU) could offer a new treatment option for highly malignant colon cancer. In our study, differential microRNA expression profiling revealed that miR-214 is downregulated in 5-FU-resistant colon cancer cells compared to normal cells. In vitro, miR-214 could sensitize non-resistant colon cancer cells and 5-FU-resistant colon cancer cellsto 5-FU. Functionally, miR-214 inhibited cell clone formation and cell growth and enhanced 5-FU-inducing cell apoptosis and caspase-3 levels. MiR-214 targeted heat shock protein 27 (Hsp27), as confirmed via dual luciferase reporter assays and western blots. Hsp27 also sensitized HT-29 and LoVo to 5-FU by enhancing cell apoptosis. Overexpression of Hsp27 could block miR-214 with an effect on the sensitivity of colon cancer cells to 5-FU. In conclusion, miR-214 sensitizes colon cancer cells to 5-FU by targeting Hsp27, indicating a significant role for this miRNA in colon cancer chemotherapy.

**Electronic supplementary material:**

The online version of this article (10.1186/s11658-019-0143-3) contains supplementary material, which is available to authorized users.

## Introduction

MicroRNAs (miRNA or miRs) are a class of endogenous, small noncoding RNAs that negatively regulate target gene expression by binding to the 3′-untranslated region (3’UTR) of mRNAs for translational repression or degradation [1, 2]. Previous studies have revealed that miRNAs are involved in various cellular processes, including cell growth, development and apoptosis, but also in the chemotherapy response [3]. MiR-214reportedly plays a role in several cancer types and has been implicated in many pathways [4, 5]. Recent studies have shown that it functions as a tumor suppressor in human colon cancer [6, 7] and can bind to the3’UTR of ARL2. MiR-214 can also target Necl-2 and regulate ErbB2/ErbB3 signaling [8].

Human colon cancer is the third leading cause of cancer death worldwide [9]. Chemotherapy resistance is a major factor in the treatment difficulty of this cancer type. For example, if resistance to the chemotherapeutic 5-fluorouracil (5-FU) could be overcome, it would give another promosing option for treating this highly malignant cancer.

Heat shock protein 27 (Hsp27) has multiple roles in colon cancer. It shows different expression levels in left-sided and right-sided colon cancers [10]. In immunogenic rats, Hsp27 was shown to enhance the tumorigenicity of colon carcinoma cell clones [11]. In colon cancer cells, Hsp27 is also involved in cell chemoresistance. Several reports have shown that Hsp27 affects their sensitivity to 5-FU.

In our study, differential microRNA expression profiling revealed that miR-214 is downregulated in 5-FU-resistant colon cancer cells compared to normal cells. The objective of this study was to determine whether miR-214 regulates the sensitivity of colon cancer cells to 5-FU by targeting Hsp27.

## Materials and methods

### Cell culture and transfection

Two colon cancer cell lines were used: HT-29 and LoVo (American Type Culture Collection; ATCC). The cells were propagated according to ATCC instructions. HT-29 cells were cultured in RPMI-1640 medium(Invitrogen) and LoVo cells in F12 medium(Invitrogen), bothsupplemented with 10% FBS(HyClone) and maintained at 37 °C with 5% CO_2_.Lipofectamine 2000 Reagent (Invitrogen) was used for transfection according to the manufacturer’s protocol. A spiked red fluorescent protein-expressing vector was used to monitor transfection efficiency.

### RNA isolation and quantitative reverse transcription PCR (qRT-PCR)

Cells were lysed with TRIzol reagent (Invitrogen) and total RNA was isolated according to the manufacturer’s instructions. The cDNA for the mRNA and miRNA was synthesized from total RNA using the Promega RT Kit. One microgram of total RNA was reverse transcribed in 50 μl using an oligo-dT primer (TaKaRa Biotechnology) and 250 ng of total RNA with an miR-100-specific stem-loop RT primer. GAPDH and U6 were used as internal controls. qRT-PCR was performed on a Realplex Real-Time PCR Detection System (Eppendorf) using SYBR Premix ExTaq reagent (TaKaRa Biotechnology) using the following conditions: 92 °C for 2 min, followed by 40 cycles of amplification at 92 °C for 30 s, and 60 °C for 1 min. The miRNA primers for reverse transcription were designed using miRNA stem-loop methods. The reverse transcriptionprimersof miRNAs were as follows: miR-203RT primer:“CTCAACTGGTGTCGTGGAGTCGGCAATTCAGTTGAGAACTGTTG”; miR-203 PCR forward primer: “ACACTCCAGCTGGGAGTGGTTCTTAA”; miR-197RT primer: “CTCAACTGGTGTCGTGGAGTCGGCAATTCAGTTGAGCCTCCCAC”; miR-197 PCR forward primer: “ACACTCCAGCTGGGCGGGTAGAGAGG”; miR-214 RT primer: “CTCAACTGGTGTCGTGGAGTCGGCAATTCAGTTGAGACTGCCTG”; miR-214 PCR forward primer: “ACACTCCAGCTGGGACAGCAGGCACA”;miR-192 RT primer:“CTCAACTGGTGTCGTGGAGTCGGCAATTCAGTTGAGGGCTGTCA”; miR-192 PCR forward primer: “ACACTCCAGCTGGGCTGACCTATGAA”;miR-605 RT primer:“CTCAACTGGTGTCGTGGAGTCGGCAATTCAGTTGAGAGGAGAAGGCAC”; miR-605 PCR forward primer: “ACACTCCAGCTGGGTAAATCCCATGG”;miR-27b RT primer: CTCAACTGGTGTCGTGGAGTCGGCAATTCAGTTGAGGCAGAACT; miR-27b PCR forward primer: “ACACTCCAGCTGGGTTCACAGTGGCT”;U6 RT primer: “CTCAACTGGTGTCGTGGAGTCGGCAATTCAGTTGAGAAAAATA”; U6 PCR forward primer: “AGAGAAGATTAGCATGGCCCCTG”; and common reverse primer: “CTCAACTGGTGTCGTGGA”. The primers for Hsp27 PCR were designed using Primer Premier 5.0 software: Hsp27 forward primer: “AGGATGGCGTGGTGGAGA” and reverse primer: “GGGAGGAGGAAACTTGGGTG”; and GAPDH forward primer: “AATGCATCCTGCACCACCAA” and reverse primer: “GTAGCCATATTCATTGTCATA”. The relative quantification of the RNA level wad calculated using the 2-^^Cq method [12].

### Construction of expression vectors

MiR-214 mimics, mimic controls, miR-214 antisense oligonucleotides (ASO) and ASO controls were all purchased from Guangdong Ribobio.Hsp27-specific small interfering RNA (Hsp27-siRNA), and thesiRNAcontrolwere purchased from GeneChem. The full length of Hsp27 was amplified and cloned into a pcDNA3.0 vector to generate an Hsp27-expression vector (pcDNA3-Hsp27), and the empty pcDNA3.0 vector was used as a control.

### CCK-8 assay

Cells were plated in 96-well plates at 4000 to 8000 cells per well and were allowed to adhere overnight. After 48 h culture with 5-FU, the IC_50_ values were determined using a Cell Counting Kit-8 (DojinDo). We added 10 μl of CCK-8 solution to each well, incubated the plates for 3.5 h in an incubator at 37 °C with 5% CO_2_, and then measured the optical densities at 450 nm using an Emax precision microplate reader (Molecular Devices).

### Colony formation assay

Cells were seeded at a density of 100 cells/well into 24-well plates. The medium was replaced every 3 days until the majority of the colonies consisted of > 50 cells. The colonies were then washed, fixed and stained using crystal violet (Sigma-Aldrich). Finally, images of the stained colonies were captured, and the colonies were counted (G16, Canon Inc.).

### TdT-mediated dUTP nick end labeling (TUNEL) assay

A TUNEL kit (Roche) was used to assess cell apoptosis following the manufacturer’s instructions. Briefly, the cells were cultured on coverslips in 24-well plates and fixed in 4% paraformaldehyde. The terminal deoxynucleotide transferase (TdT) reaction was performed for 1 h at 37 °C in a humidified chamber. Then, the cells were stained with 4,6-diamidino-2-phenylindole (DAPI) for 5 min. The numbers of TUNEL-positive cells and the total cells were counted under a fluorescence microscope. For the analysis of apoptosis, the percentage of apoptotic cells (apoptosis ratio) was determined as the positive cell number/total cell number × 100. TUNEL-positive cells and cell nuclei respectively exhibited green fluorescence and blue fluorescence.

### Luciferase reporter assay

The 3’UTR of Hsp27 was amplified and inserted downstream of a luciferase reporter construct. Mutant 3’UTRs of Hsp27 were amplified using wild-type Hsp27 3’UTR as the template. Cells were cotransfected with miR-214 ASO or miR-214 mimics and the luciferase reporter construct. After transfection for 48 h, cells were collected and lysed with RIPA buffer. Luciferase intensity was measured using the Dual-Luciferase Reporter Assay System (Promega Corporation) according to the manufacturer’s instructions.

## Western blot

Cells were washed twice with phosphate buffered saline(PBS)and lysed directly in lysis buffer consisting of 50 mM Tris-HCl (pH 8.8), 150 mM NaCl, 1% NP-40, 1% sodium deoxycholate and 0.1% SDS. The protein concentration of the lysate was measured using a Bradford protein assay kit (Bio-Rad). A total of 20 μg of protein was used for the analysis of Hsp27 expression and GAPDH was used as a loading control. The primary antibodies were used as follows:rabbit polyclonal to Hsp27 (ab78806, 1:500), rabbit polyclonal toGAPDH (ab9485,1:1000), rabbit polyclonal to Hsp27(phospho S86, ab17938,1:500), mouse monoclonal toP38 MAPK(ab31828,1:500), and rabbit polyclonal to P38 MAPK(phosphoY182, ab47363, 1:500). All antibodies were purchased from Abcam. Goat anti-rabbit or anti-mouse immunoglobulin G conjugated with horseradish peroxidase (1:5000) were used as the secondary antibodies. Proteins were visualized using enhanced chemiluminescence reagent and were detected with a Western Blotting Detection System (GE Healthcare Bio-Sciences).

### Statistical analysis

All experiments were replicated three times, and the data are presented as the means ± SEM. Statistical significance was analyzed using the SPSS 17.0 software package. Student’s t-test was used for analysis of the difference between two groups, while one-way ANOVA wad used to analyse differences between multiple groups. *p* < 0.05 was considered as a significant difference.

## Results

### MiR-214 sensitized colon cancer cells to 5-FU

**Fig. 1:**
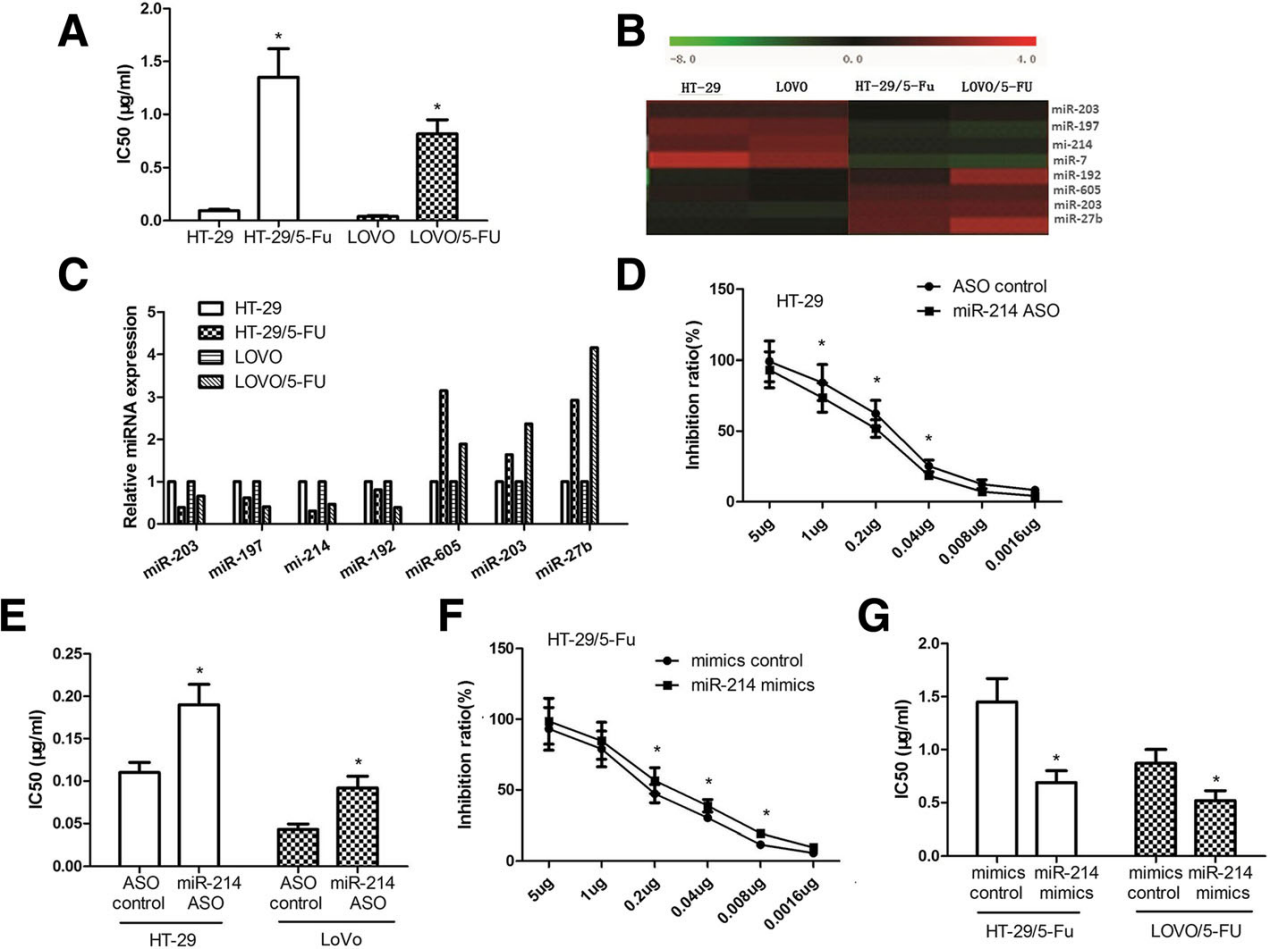
miR-214 sensitizes colon cancer cells to 5-FU. **a** – IC_50_ values for 5-FU from HT-29/5-FU, LoVo/5-FU and their parental cells were determined using the CCK-8 assay. **b** – The differential miRNA expressional profiles from HT-29, HT-29/5-FU, LoVo and LoVo/5-FU cells determined using microarray assays. **c** –Quantification of miRNA expression inHT-29, HT-29/5-FU, LoVo and LoVo/5-FU cells was performed via qPCR to validate the results of the microarrays. **d** and **e** – HT-29 and LoVo cells were both transfected with miR-214 ASO and control. After incubation with 5-FU at concentrations of 0.0016, 0.008, 0.04, 0.2 and 1.5 μg/ml for 48 h, inhibitions were detected using CCK-8 assays, and the IC_50_ values were determined. F and G – HT-29/5-FU and LoVo/5-FU cells were both transfected with miR-214 mimics and controls. Inhibitions (**f**) and IC_50_ values (**g**) were determined as in **d** and **e**. Data represent the means ± SEM of three independent experiments. *p < 0.05

We successfully constructed 5-FU-resistant colon cancer cell lines HT-29/5-FU and LoVo/5-FU. The 50% inhibitory concentration (IC_50_) of 5-FU for HT-29/5-FU and LoVo/5-FU were both significantly higher than for their parental cells (Fig. 1a; *p* < 0.05). The miRNA expression profile revealed that miR-214 was downregulated in 5-FU-resistant cells compared to parental cells (Fig. 1b and c). In HT-29 and LoVo cells, inhibition of miR-214 bytransfection with miRNA ASO reduced the cell growth inhibition by 5-FU and enhanced the IC_50_ of the cells (Fig. 1d and e).In HT-29/5-FU and LoVo/5-FU, overexpression of miR-214 by transfection with miR-214 mimics enhanced the cell growth inhibition of 5-FU and reduced the IC_50_ of cells (Fig. 1f and g; *p <* 0.05).These results indicated that miR-214 sensitized the colon cancer cells to 5-FU.

### MiR-214 sensitized colon cancer cells to 5-FU by activating caspase-3

**Fig. 2:**
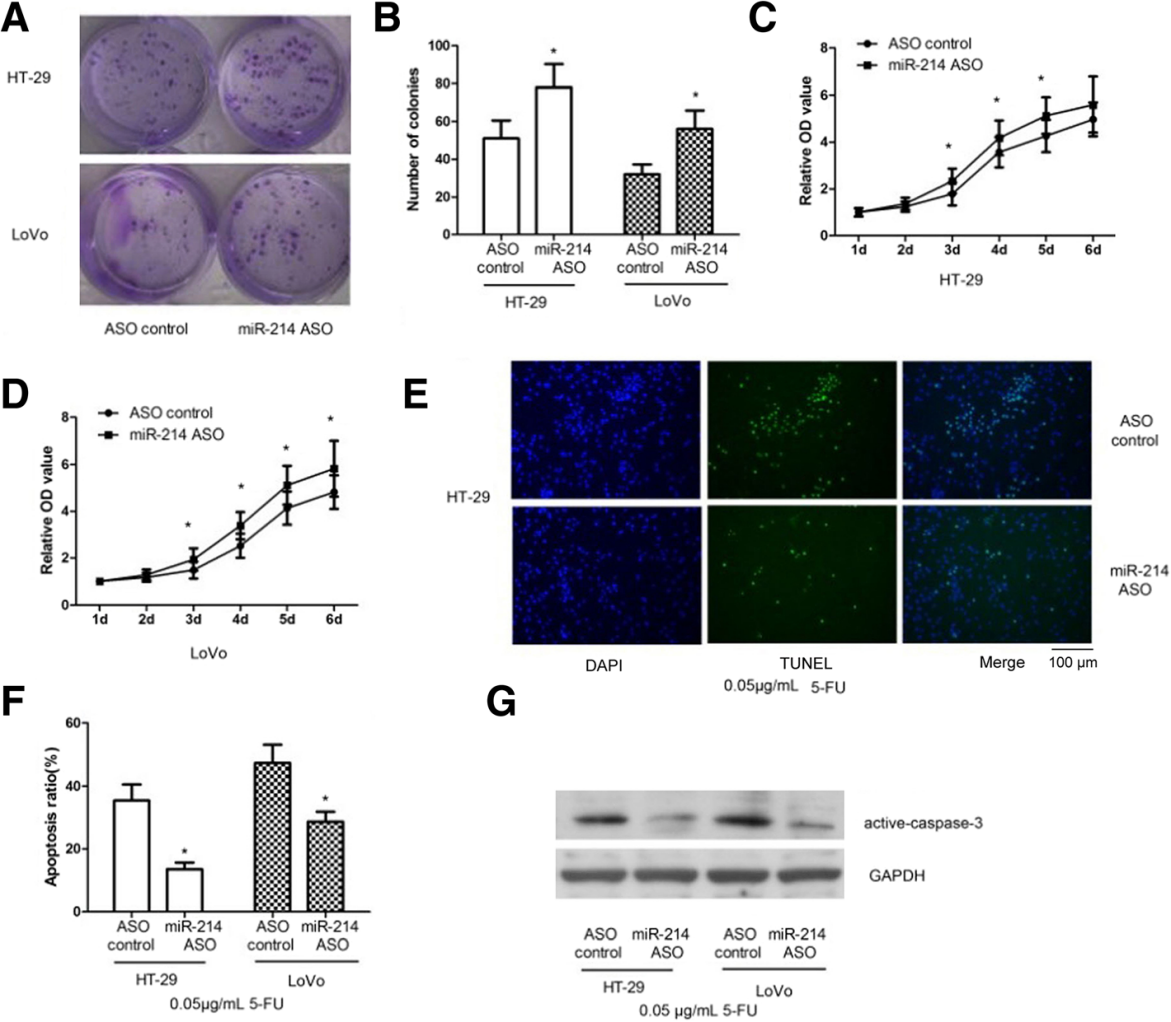
miR-214 sensitizes colon cancer cells to 5-FU by activating caspase-3. **a** and **b** –HT-29 and LoVo cells were transfected with miR-214 ASO and ASO control and harvested 48 h after transfection. Colony formation assays were done to detect cell growth after 14 days of culture. **c** and **d** –Relative OD values were determined using CCK-8 assays after 1, 2, 3, 4, 5 and 6 days in HT-29 and LoVo cells. **e** and **f** – Cells were incubated with 0.05 μg/ml of 5-FU for 48 h. TUNEL assays were performed to detect cell apoptosis. The blue stain is the cell nucleus; green is TUNEL staining representing apoptotic cells (scale bar = 100 μm). **g** – Western blots were done to detect caspase-3 protein levels. Data represent the means ± SEM from three independentexperiments. *p < 0.05

HT-29 and LoVo cells were transfected with miR-214 ASO and ASO control. After 14 days of culture, colony-formation assays showed that inhibition of miR-214 promoted cell colony formation (Fig. 2a and b; *p <* 0.05). After 6 days of detection, the cell growth curves showed an inhibition of miR-214-promoted cell growth (Fig. 2c and d). After a 48 h culture with 0.05 μg/ml 5-FU, TUNEL assays showed inhibition of miR-214 and significantly lower cell apoptosis (Fig. 2e and f; *p <* 0.05). The results of western blotting revealed that a knockdown of miR-214 reduced the 5-FU-inducing active caspase-3 protein level (Fig. 2g and Additional file 1). These results indicate that miR-214 may sensitize colon cancer cells to 5-FU by activating caspase-3.

### MiR-214 could target Hsp27 by binding to its 3’UTR

**Fig. 3:**
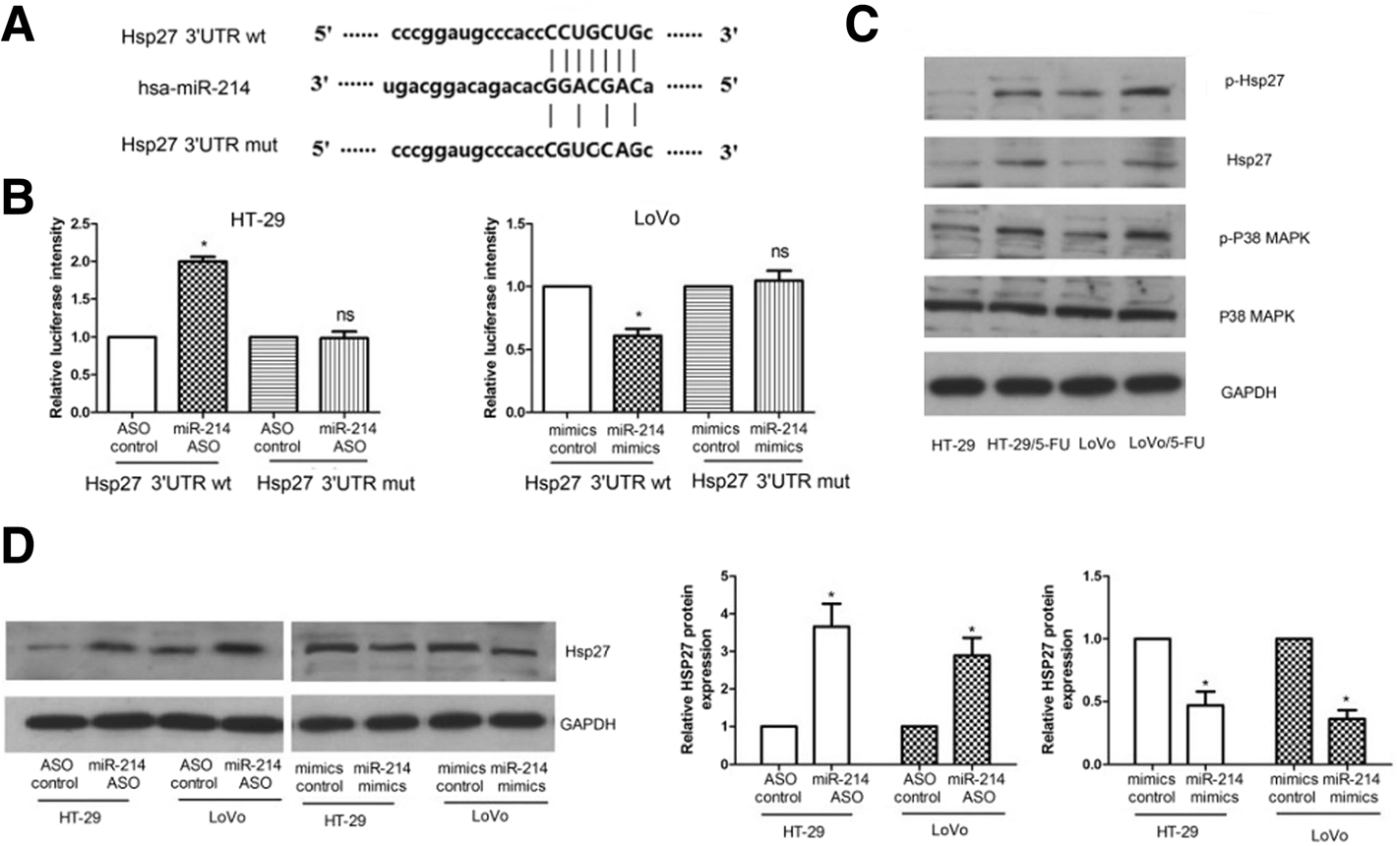
miR-214 targets Hsp27 by binding to the 3’UTR. **a** – The complementary site of miR-214 in the 3’UTR of Hsp27 as predicated using bioinformatic analysis. **b** – Hsp27 3’UTR wtand Hsp27 3’UTR mutant luciferase constructs were simultaneously transfected into cells with miR-214 ASO, miR-214 mimics or corresponding controls. Luciferase activities were measured at 48 h post-transfection. **c** – Western blots were done to detect the protein levels of Hsp27, p-Hsp27, p-P38 MAPK and P38 MAPK in the cells. GAPDH was used as the internal control protein. **d** – Hsp27 protein levels in miR-214 ASO or miR-214 mimics transfected into HT-29 and LoVo cells were measured viawestern blot. Data represent the means ± SEM from three independentexperiments. *p < 0.05

The bioinformatics BLAST showed that miR-214 could bind to the 3’UTR of Hsp27. We constructed two types of luciferase reporter constructs, including a wild-type or mutant-type 3’UTR of Hsp27 (Fig. 3a). Luciferase reporter assays showed that miR-214 ASO significantly increased the firefly luciferase activity of the vector with the wild-type 3’UTR of Hsp27 cells but had no significant effect on the vector with the mutant 3’UTR of Hsp27 in HT-29 and LoVo cells (Fig. 3b). These results indicate that miR-214 could bind to the sequence of the Hsp27 3’UTR. In HT-29 and LoVo cells, western blotting showed that miR-214 could negatively regulate Hsp27 protein levels (Fig. 3d and e). Detection of the protein levels in cells showed that Hsp27, p-Hsp27, P38 MAPKand p-P38 MAPK were upregulated in 5-FU-resistant cells compared to the parental cells (Fig. 3c and Additional file 1). These data indicate that Hsp27 is a target of miR-214 and may be involved with cell sensitivity to 5-FU.

### Hsp27 couldaffect thesensitivity of colon cancer cells to 5-FU

**Fig. 4:**
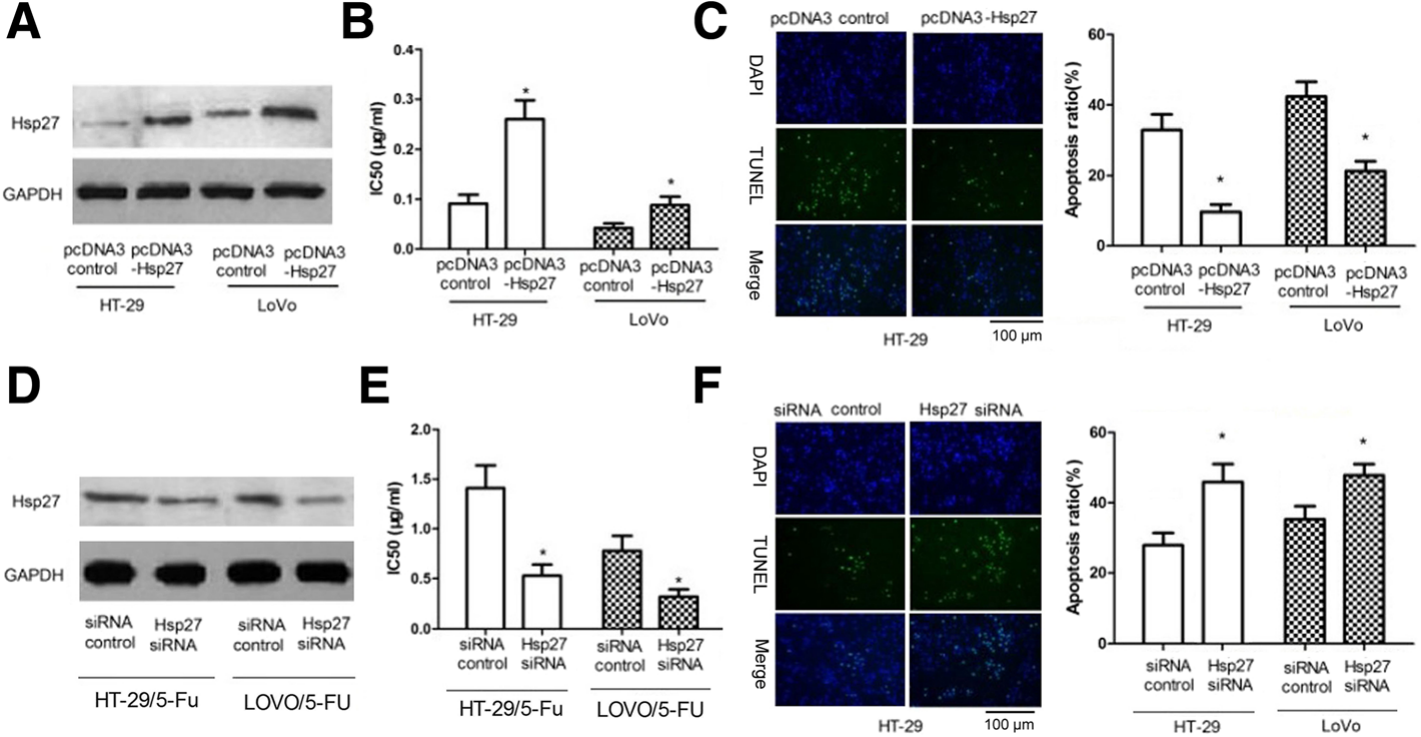
Hsp27 affects the sensitivity of colon cancer cells to 5-FU. **a**, **b** and **c** – HT-29 and LoVo cells were transfected with pcDNA3-Hsp27 or pcDNA3 controls. Hsp27 protein expression was detected at 48 h post-transfection via western blot (**a**). After cells were incubated with 5-FU at concentrations of 0.0016, 0.008, 0.04, 0.2 and 1.5 μg/ml for 48 h,IC_50_ values were determined using CCK-8 assays (**b**). After the cells were incubated with 0.05 μg/ml 5-FU for 48 h, cell apoptosiswas determined using TUNEL assays (**c**, scale bar = 100 μm). **d**, **e** and **f** – HT-29 and LoVo cells were transfected with Hsp27 siRNA or siRNA controls. Hsp27 protein expression (**d**), IC_50_ (**e**) and cell apoptosis (**f**, scale bar = 100 μm) were assessed. Data represent the means ± SEM from three independentexperiments. *p < 0.05

We successfully constructed colon cancer cell lines that overexpressed Hsp27 by transfecting pcDNA3-Hsp27 into the cells. Western blotting confirmed the Hsp27 overexpression (Fig. 4a). The results of the IC_50_ assays showed that overexpression of Hsp27 enhanced cell resistanceto 5-FU (Fig. 4b; *p <* 0.05). After a 48 h culture with 0.05 μg/ml5-FU, TUNEL assays showed that overexpression of Hsp27 reduced5-FU-inducing cell apoptosis (Fig. 4c). Knockdowns of Hsp27 were constructed by transfection with Hsp27 siRNA and western blots showed a knockdown of Hsp27 (Fig. 4d and Additional file 1). The results of the IC_50_ assays showed that a knockdown of Hsp27 significantly sensitized the colon cancer cells to 5-FU (Fig. 4e). After a 48 h culture with 0.05 μg/ml5-FU, TUNEL assays showed that the knockdown of Hsp27 enhanced5-FU-inducing cell apoptosis (Fig. 4f). These results indicate that Hsp27 could affect the sensitivity of colon cancer cells to 5-FU.

### Hsp27 could block the effect of miR-214

**Fig. 5:**
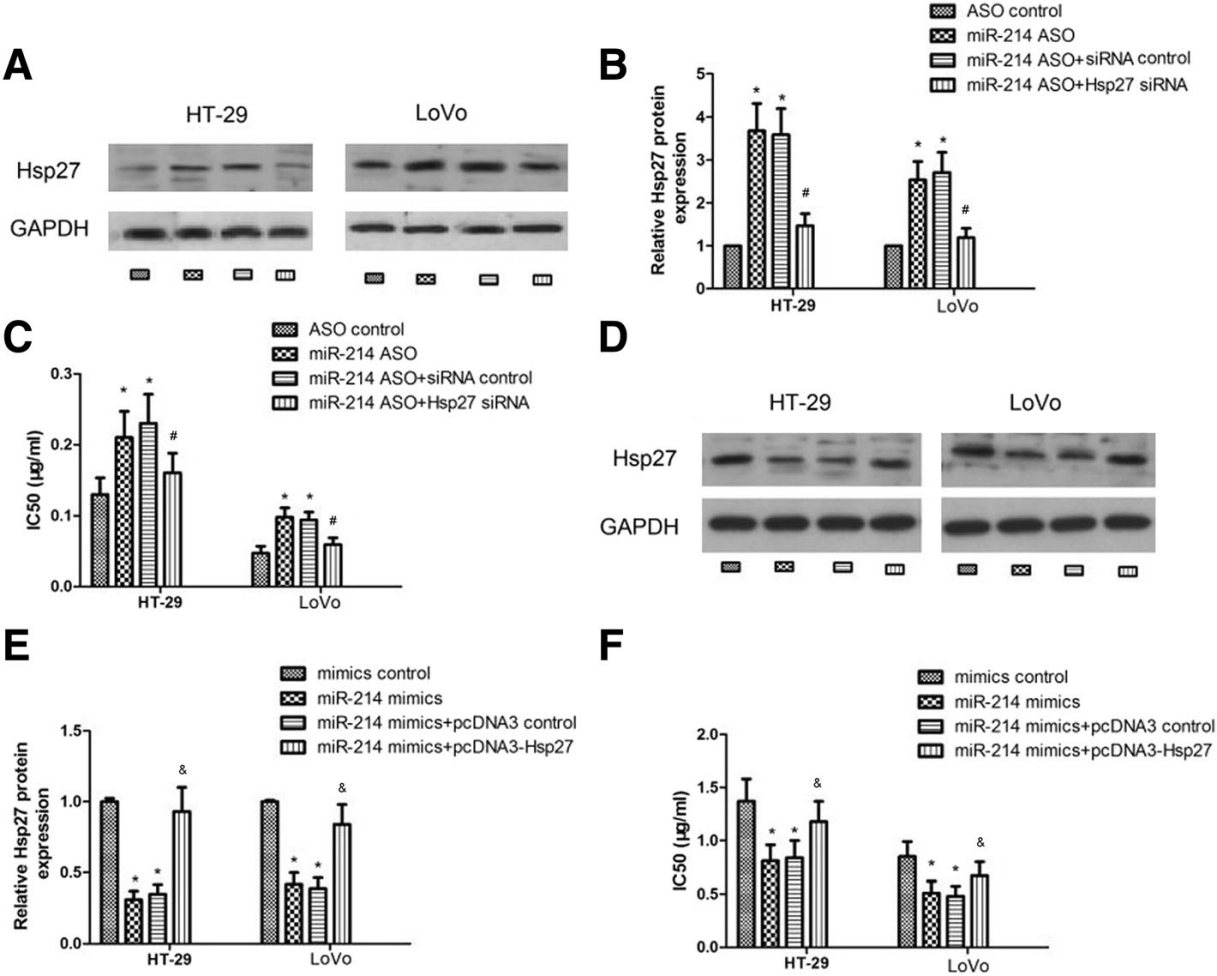
Hsp27 blocks the effect of miR-214. **a**, **b** and **c** – HT-29 and LoVo cells were transfected with ASO control, miR-214 ASO, miR-214 ASO + siRNA control or miR-214 ASO + Hsp27 siRNA. Protein expression of Hsp27 was detected via western blot (**a** and **b**) and IC_50_ values were detected using CCK-8 assays (**c**). **d**, **e** and **f** –HT-29 and LoVo cells were transfected with mimics control, miR-214 mimics, miR-214 mimics+pcDNA3 control or miR-214 mimics+pcDNA3-Hsp27.Protein expression of Hsp27 was detected via western blot (**d** and **e**) and IC_50_ values were detected using CCK-8 assays (**f**). Data represent the means ± SEM from three independentexperiments. *compared to control, *p <* 0.05;^#^compared tomiR-214 ASO + siRNA control, *p <* 0.05; ^&^compared to miR-214 mimics+pcDNA3 control, *p <* 0.05

To further investigate the relationship between miR-214 and Hsp27,we transfected Hsp27 siRNA into cells with miR-214 ASO and then performed IC_50_ assays. As shown by western blotting (Fig. 5a and b) and IC_50_ assays (Fig. 5c), inhibition of miR-214 after the transfection of siRNA controls could still enhance cell resistance to 5-FU, but inhibition of miR-214 could no longer enhance cell resistance to 5-FU after Hsp27 knockdown. By contrast, overexpression of Hsp27 could also block the effectof miR-214overexpression (Fig. 5d-f; *p <* 0.05). These data all indicate that miR-214 sensitizes colon cancer cells to 5-FU by targeting Hsp27.

## Discussion

MiRNAs play an important role in the regulation of protein-coding gene expression and exhibit a crucial function in cellular processes [13]. Accumulating evidence shows that abnormal expression of miRNAs is associated with cancer initiation and response to treatment [14]. Abnormal expression of miR-214 is found in many types of cancer. In hepatoma, miR-214 is downregulated and inhibits tumor angiogenesis by inducing hepatoma-derived growth factor. A previous study showed that miR-214 expression is decreased in human colon cancer and that it inhibited cell proliferation and induced cell apoptosis [6, 7].

Our results concur: the inhibition of miR-214 inhibited cell colony formation and affected cell growth curves. We also found that the level of miR-214 was related to cell sensitivity to 5-FU. In 5-FU-resistant colon cancer cells, miR-214 had lower expression levels. Inhibiting miR-214 enhanced the cells’ resistance to 5-FU.

Currently, there are no other studies exploring the relationship between 5-FU and miR-214 levels in colon cancer. In certain other cancers, including ovarian cancer, miR-214 has also been shown to function as a tumor suppressor and to induce cell survival and cisplatin resistance by targeting the phosphate and tensin homolog 3’UTR [6]. In addition, miR-214 has been found to be upregulated in many other cancer types, including gastric cancer [15, 16] and liver cancer [17]. Overexpression of miR-214 enhanced tumor malignance. These results indicate that miR-214 plays different roles in different types of tissues and cells.

Research also shows that miR-214 expression is related to the chemotherapy response. Expression of miR-483 and miR-214 induced multidrug resistance in esophageal squamous cell carcinoma [18]. In EGFR mutant cell lines, suppression of miR-214 may reverse their acquired resistance to EGFR-TKI therapies [19]. These data indicate that mi-214 has a role in cancer chemotherapy. Our data indicate that the inhibition of miR-214 enhanced the resistance of colon cancer cells to 5-FU.

Heat shock protein 27 (HSP27) is located on chromosome 7 and encodes a 27-kDa protein with an α-crystallin domain [20]. Normally, Hsp27 protects cells from stress and is associated with cell motility and cytoskeletal stabilization [21, 22]. Abnormal expression of Hsp27 plays an important role in cancer genesis and development. Hsp27 expression also plays an important role in colon cancer [23]. Bioinformatic analysis has shown that HSP27 is a candidate target gene of miR-214. Our data indicate that overexpression of Hsp27 could sensitize colon cancer cells to 5-FU. These results are in accordance with the reported data that the inhibition of Hsp27 sensitized colorectal cancer cells [24, 25]. MiR-214 can bind to the 3’UTR of HSP27. Overexpression of miR-214 sensitizes colon cancer cells to 5-FU by targeting Hsp27.

## Conclusions

We have demonstrated that miR-214 expression decreases in 5-FU-resistant colon cancer and that the knockdown of miR-214 can significantly induce cell resistance to 5-FU by targeting HSP27. These findings have not been previously reported. Our data suggest that miR-214 has potential as a therapeutic agent for the treatment of colon cancer.

## Additional file

Additional file 1: Quantified Data. (TIF 892 kb)

## Abbreviations

3’UTR: 3′ untranslated region
5-FU: 5-fluorouracil
ASO: antisense oligonucleotides
Hsp27: heat shock protein 27
IC_50_: 50% inhibitory concentration
PBS: Phosphate-buffered saline
qPCR: quantitative real-time polymerase chain reaction
siRNA: small interfering RNA
TUNEL: TdT-mediated dUTP nick end labeling
