# Supplementary figures and images for: MiR-214 sensitizes human colon cancer cells to 5-FU by targeting Hsp27
Source: Cell Mol Biol Lett. 2019 Mar 14;24:22. doi: 10.1186/s11658-019-0143-3 (PMC6419349; doi:10.1186/s11658-019-0143-3)

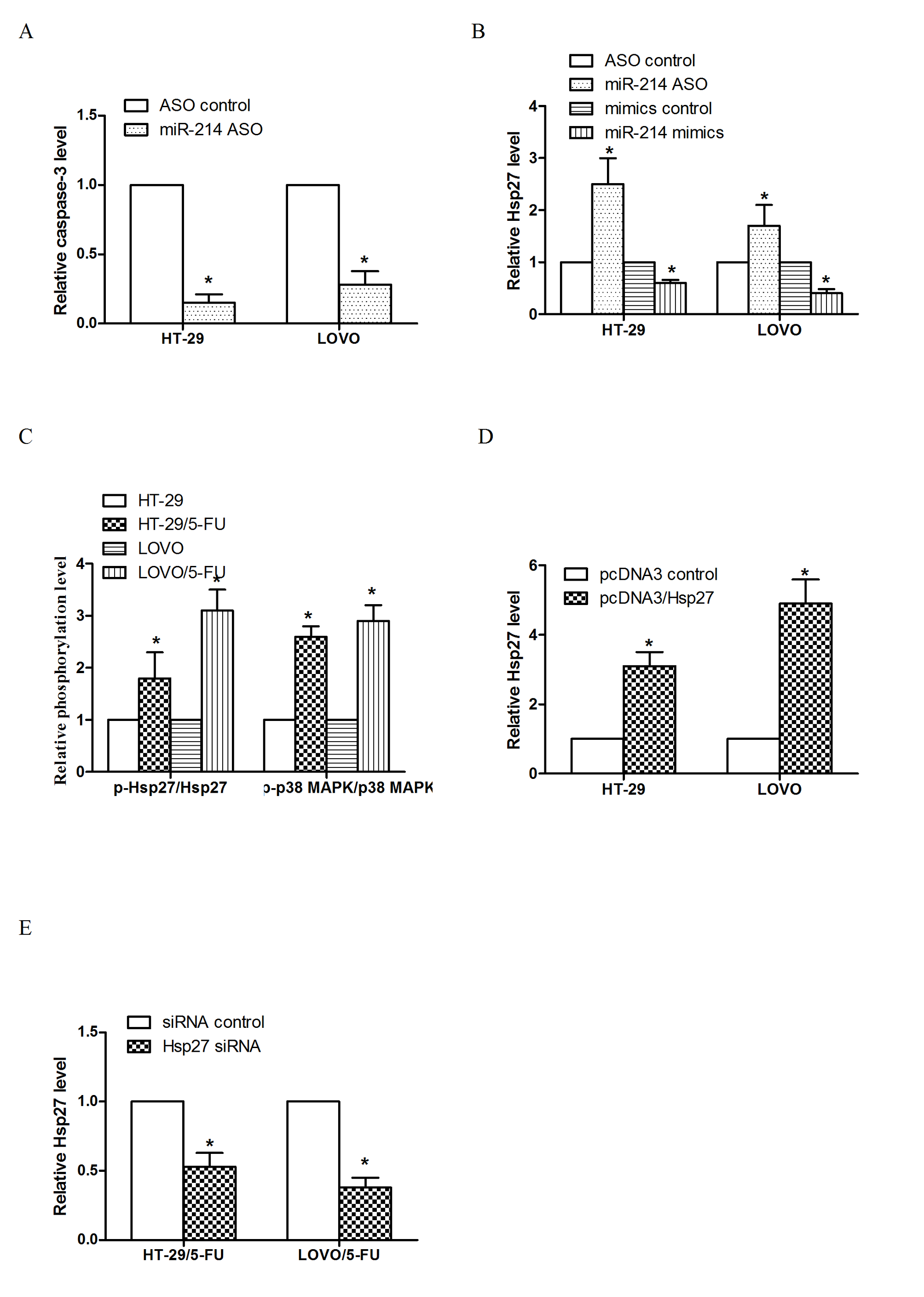

Supplement: Supplementary file 1 — Quantified Data. (TIF 892 kb) [file 11658_2019_143_MOESM1_ESM.tif]
